# Machine Learning to Identify Patients at Risk of Developing New-Onset Atrial Fibrillation after Coronary Artery Bypass

**DOI:** 10.3390/jcdd10020082

**Published:** 2023-02-15

**Authors:** Orlando Parise, Gianmarco Parise, Akshayaa Vaidyanathan, Mariaelena Occhipinti, Ali Gharaviri, Cecilia Tetta, Elham Bidar, Bart Maesen, Jos G. Maessen, Mark La Meir, Sandro Gelsomino

**Affiliations:** 1 Cardiovascular Research Institute Maastricht (CARIM), Maastricht University Medical Centre, Universiteitssingel 50, 6229 ER Maastricht, The Netherlands; 2 Department of Cardiac Surgery, Universitair Ziekenhuis Brussel, Laarbeeklaan 101, 1090 Brussels, Belgium; 3 Radiomics, 4000 Liège, Belgium; 4 Institute of Computational Science, Università della Svizzera Italiana, 6900 Lugano, Switzerland

**Keywords:** machine learning, artificial intelligence, atrial fibrillation, postoperative CABG

## Abstract

Background: This study aims to get an effective machine learning (ML) prediction model of new-onset postoperative atrial fibrillation (POAF) following coronary artery bypass grafting (CABG) and to highlight the most relevant clinical factors. Methods: Four ML algorithms were employed to analyze 394 patients undergoing CABG, and their performances were compared: Multivariate Adaptive Regression Spline, Neural Network, Random Forest, and Support Vector Machine. Each algorithm was applied to the training data set to choose the most important features and to build a predictive model. The better performance for each model was obtained by a hyperparameters search, and the Receiver Operating Characteristic Area Under the Curve metric was selected to choose the best model. The best instances of each model were fed with the test data set, and some metrics were generated to assess the performance of the models on the unseen data set. A traditional logistic regression was also performed to be compared with the machine learning models. Results: Random Forest model showed the best performance, and the top five predictive features included age, preoperative creatinine values, time of aortic cross-clamping, body surface area, and Logistic Euro-Score. Conclusions: The use of ML for clinical predictions requires an accurate evaluation of the models and their hyperparameters. Random Forest outperformed all other models in the clinical prediction of POAF following CABG.

## 1. Introduction

Atrial fibrillation is the most common supraventricular arrhythmia [1,2], and its incidence is dramatically rising worldwide [3]. The number of people with atrial fibrillation (AF) in Europe is expected to double to >17 million by 2060 due to aging populations [4]. Therefore, AF can be considered a 21st-century “cardiovascular disease global epidemic” due to its dramatic medical, social, and economic burden [5,6,7,8,9].

The results after either interventional or pharmacological treatment are suboptimal regarding stable-long-term patient freedom from atrial fibrillation [10,11,12,13].

The high incidence of AF recurrence after the treatment imposes a substantial extra burden on the healthcare system due to increased morbidity, mortality, associated therapeutic interventions, and other costs such as patient visits, anticoagulation status, and side effects from drug therapy [14]. Therefore, there is a general agreement amongst experts that there is a pressing need to improve AF treatment [15].

Stroke and thromboembolism are the main complications of AF [16,17]. However, AF can also be associated with heart failure, arterial hypertension, diabetes, and valve disease, posing a significant burden to patients, physicians, and healthcare systems [18,19]. Furthermore, surgery can also trigger AF, representing the so-called new-onset postoperative atrial fibrillation (POAF), which complicates 20–40% of cardiac surgical procedures and 10–20% of non-cardiac thoracic operations [5,7,8,9,10,11]. POAF is also the most common arrhythmia after coronary artery bypass grafting (CABG) [20], and it significantly increases morbidity as well as short and long-term mortality [21,22,23,24].

Risk factors for developing POAF after CABG have been identified in several works [25], including general parameters of patients’ functional status (older age, low ejection fraction, comorbidities such as chronic obstructive pulmonary disease and chronic renal dysfunction) as well as more specific parameters such as preoperative withdrawal of beta-blocker drugs. However, in most of the available literature, only classical statistical methods have been employed that assume linear relationships between variables and predicted outcomes. They must specify interactions between variables a priori [26].

Recently machine learning (ML) algorithms have been applied in various fields of healthcare [27], having the advantage of identifying non-linear associations between covariates and being able to predict and detect interactions between variables from observed data [28]. Nonetheless, ML algorithms have also been used for predicting the risk of developing POAF only in a few papers and in very small cohorts [29].

This study aims to develop an effective prediction model of POAF following CABG operations and to highlight the most relevant patient and clinical features involved through ML algorithms.

## 2. Materials and Methods

### 2.1. Data Source, Patients Selection, and Definitions

This retrospective study includes three hundred ninety-four patients undergoing CABG at the Cardiothoracic Department (CTC) of Maastricht University Medical Center+ (MUMC+) between 2010 and 2017.

The study included patients above 18 years old undergoing first-time CABG. Patients who had previous cardiac surgery were excluded, as well as those with documented AF or who received anti-coagulant therapy within six months before CABG. No other exclusion criteria were applied.

POAF was defined as an acute or new-onset episode with irregular RR-intervals in an electrocardiogram (ECG) without a traceable p-wave for at least 10 s [30,31,32] and occurring during the postoperative period in-hospital stay.

After excluding underlying medical comorbidities like electrolyte imbalance, amiodarone was started (2.5–5 mg/kg IV over 20 min, then 15 mg/kg). Electrical cardioversion was employed in case of a failed pharmacological attempt, POAF lasting over 48 h, or hemodynamic instability.

### 2.2. Variables and Preliminary Analysis

Variables included demographic characteristics, laboratory data related to renal function, surgical parameters, and postoperative complications. The Logistic Euro-Score was employed, which is largely used in cardiac surgery patients for individual risk prediction, including the very high-risk patient.

A preliminary analysis was performed on variables with zero- and near-zero-variance features (i.e., they had a single value or a handful of unique values that occurred with very low frequencies) that may cause a model to fail or the fit to be unstable [33]. These variables were merged into a single variable to bypass the abovementioned issue.

### 2.3. Data Pre-Processing

Before running ML procedures, some pre-processing steps were carried on.

The dataset was split in two at a 75:25 ratio, a training dataset (296 patients) was used to feed the models, and a held-out test dataset (96 patients) was used to assess the performance of the models. The splitting process kept the postoperative AF/non-AF ratio consistent between the datasets.

Numeric variables were centered (subtracted by mean), scaled (divided by standard deviation), and normalized in the range 0 to 1. Categorical variables were one-hot-encoded (each class was created in the un-coded variable). The prediction of missing preoperative creatinine values (the only variable with missing values, 10.1%) was performed by the k-nearest neighbors’ method.

### 2.4. Machine Learning Algorithms

Four ML algorithms were employed, and their performances were compared: Multivariate Adaptive Regression Spline (MARS), Neural Network (NN) with three hidden layers, Random Forest (RF), and Support Vector Machine (SVM) with a radial basis kernel function. After the built-in features selection, each algorithm was applied to the training dataset to build the predictive model. The following functions were called to create the models: “earth” with MARS, “mlpMl” with NN, “rf” with RF, and “svmRadial” with SVM.

A hyperparameters search was adopted to optimize each model for better performance, and the Receiver Operating Characteristic (ROC) Area Under the Curve (AUC) metric was selected to choose the best model. The search consisted of two phases: a preliminary step to detect a plausible set of values and a grid criteria step to fine-tune the editable hyperparameters in the functions used.

The hyperparameters taken into account by the individual models were as follows: with MARS, nprune (maximum number of terms (including the intercept) in the pruned model) and degree (maximum degree of interaction); with NN, the size of the three hidden layers; with RF, mtry (number of variables randomly sampled as candidates at each split); with SVM, sigma (inverse kernel width) and cost (cost regularization parameter, it controls the smoothness of the fitted function—higher values lead to less smooth functions).

A 10-fold cross-validation method was set during the training step as a resampling method to validate each model. Furthermore, the importance of the variables was estimated. An up-sampling technique (randomly replicated instances, with replacement, in the minority class) was employed during the training step to address the class imbalance in the training dataset. Moreover, ROC AUC, sensitivity, and specificity resampling result differences between models were estimated.

Finally, each model’s best instances (according to the chosen metric, ROC AUC) were fed with the held-out test dataset (the learning steps were never applied to this data). For each prediction, a confusion matrix was generated. The following values were calculated: accuracy (true positive and true negative cases divided by all cases), sensitivity or recall (true positive cases divided by positive reference events), specificity (true negative cases divided by negative reference events), precision or positive predicted value (true positive divided by predicted positive events), negative predicted value (true negative divided by predicted negative events), F1 value (harmonic mean of precision and recall values).

Lastly, all tested models’ ROC and Precision-Recall (PR) curves were processed. The PR curve is typically employed for assessing model performances when the outcome class is very unbalanced (for example, 1% vs. 99%). We adopted the PR curve even if the outcome class of our study is not that overly unbalanced (10.2% POAF vs. 89.8% non-POAF).

Finally, as age is a well-known independent factor for AF initiation, the machine learning algorithms were trained only on the age feature to predict POAF events. The predictive power (ROC AUC) was compared with the models trained on the other features.

The analysis was carried out using R Core Team (2021) (R: A language and environment for statistical computing, version 4.1.2. R Foundation for Statistical Computing, Vienna, Austria); and by caret package, version 6.0–86 [33].

### 2.5. Traditional Logistic Regression

For comparison purposes only, the logistic regression was also calculated on the same training data, and then the accuracy was verified on the test data.

### 2.6. Statistical Significance

We assumed a statistically significant *p*-value < 0.05.

### 2.7. Generic

This work has involved various professionals, including surgeons and cardiologists fully committed to diagnosing and treating AF, general physicians, data scientists, machine learning researchers, and modeling experts [34,35,36,37,38,39,40,41].

## 3. Results

### 3.1. Pre-Processing

Table 1 summarizes demographic, clinical, and surgical data. The incidence of POAF was 10.6% (42/394). The median age was 60 years (Q1, Q3 (54, 67)), and three hundred thirty-four (84.8%) patients were male. Furthermore, patients with POAF were significantly older (*p* < 0.001) and showed a higher operative risk score (*p* = 0.013).

After splitting the initial data set in two, the training dataset had 296 cases; the test data set had 98 cases. Each dataset had the same initial value of POAF/non-POAF patient ratio. In the supplementary material, Tables S1 and S2 report the descriptive statistics of training and test datasets, respectively.

### 3.2. Machine Learning

#### 3.2.1. Analysis of Single Models

**Figure 1:**
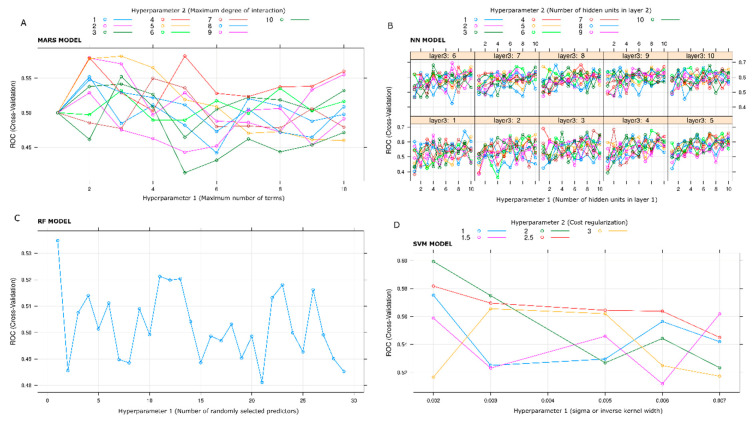
Iterations of hyperparameters search performed with MARS (**A**), NN (**B**), RF (**C**), and SVM (**D**) models after the last run, respectively. The graphs report the variations of the ROC AUC as a function of one or more parameters of each model. With the MARS model, the maximum ROC AUC value is obtained by degree and term hyperparameters equal to 4 and 5, respectively (**A**). With the NN model, the maximum ROC AUC value is obtained by layer1, layer2, and layer3 hyperparameters equal to 7, 9, and 6, respectively (**B**). With the RF model, the maximum ROC AUC value is obtained by predictors hyperparameter numbers equal to 1 (**C**). With the SVM model, the maximum ROC AUC value is obtained by sigma and cost hyperparameters equal to 0.002 and 2, respectively (**D**).

Figure 1A–D shows the various iterations of the hyperparameter search performed with MARS, NN, RF, and SVM models after the last run. The graphs report the variations of the ROC AUC as a function of one or more parameters of each model. The maximum value for the chosen metric (ROC AUC) was caught for each model, and the correspondent best model was saved as follows: with MARS, nprune = 5 and degree = 4; with NN, layer1 = 7, layer2 = 9, layer3 = 6; with RF, mtry = 1; with SVM, sigma = 0.002 and cost = 2.

Table 2 shows the obtained maximum values of ROC AUC, sensitivity, and specificity resampling values for each model. The maximum ROC AUC value (0.95) was obtained by using the SVM model, the maximum sensitivity value (1) by using the MARS, NN, and SVM models, and the maximum specificity value (1) by using the NN model.

Table 3 shows the estimated differences between the metrics values reported in Table 2 and the *p*-value (Bonferroni adjustment). ROC AUC does not show any significant difference. At the same time, sensitivity yields a statistically significant value (0.50) with (NN—RF) (*p*-value, 0.03), and specificity yields a statistically significant value (−0.19) with (MARS—RF) (*p*-value, 0.05).

**Figure 2:**
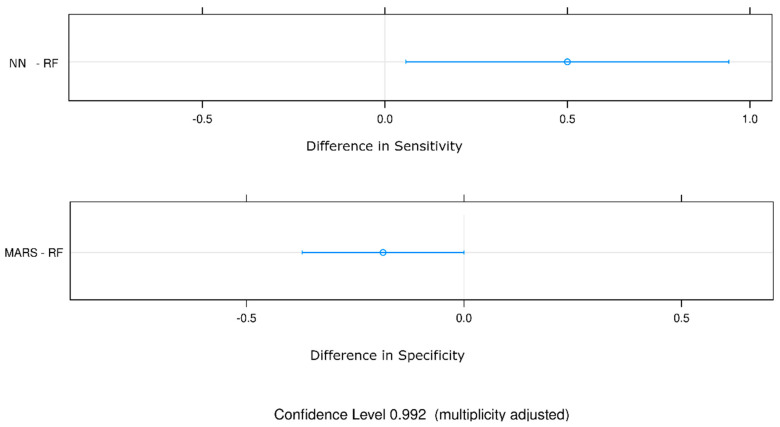
Statistically significant estimated differences of the metrics values with confidence intervals and a vertical line indicating the points with zero difference. The most statistically significant difference in sensitivity and specificity is between the NN and RF and MARS and RF models.

Figure 2 additionally shows confidence intervals of the only statistically significant differences.

#### 3.2.2. Features Selected by the Models

**Figure 3:**
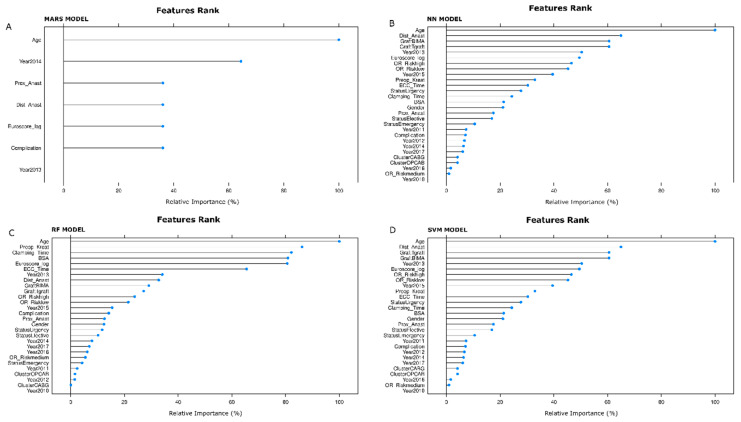
Relevant features selected by single models: MARS (**A**). NN (**B**). RF (**C**). SVM (**D**). The relevant features rank has to be interpreted from the top (most important) to the bottom (less important). An acceptable cut-off value is 35%.

Relevant features selected by MARS, NN, RF, and SVM algorithms are displayed in Figure 3A, Figure 3B, Figure 3C, and Figure 3D, respectively. The measures of importance are scaled to have a maximum value of 100. With the MARS model, the top five features were age (100.0%), Operation Year 2014 (64.4%), complications (36.1%), number of distal anastomoses (36.1%), Logistic Euro-Score (36.1%).

Using the NN model, the top five features were age (100.0%), the number of distal anastomoses (65.0%), use of double mammary artery (60.6%), the performance of T-graft anastomosis (60.6%), and the Operation Year 2013 (50.4%).

Employing the RF model, the top five features were age (100.0%), preoperative creatinine values (86.1%), time of aortic cross-clamping (82.2%), body surface area (80.9%), and Logistic Euro-Score (80.7%).

Finally, with the SVM model, the top five features were age (100.0%), the number of distal anastomoses (65.0%), use of double mammary artery (60.6%), the performance of T-graft anastomosis (60.5%), and Operation Year 2013 (50.4%).

#### 3.2.3. Testing

After feeding the models with the test data set, the confusion matrices were calculated from the predicted values (at 0.5 classification threshold) and the true outcome values.

Table 4 shows metrics values from the confusion matrices: accuracy, sensitivity (or recall), specificity, precision (or positive predictive value), negative predictive value, and F1 values for each model. RF model reaches the maximum values for all parameters, but the sensitivity for which the maximum value (0.70) is associated with the MARS model.

**Figure 4:**
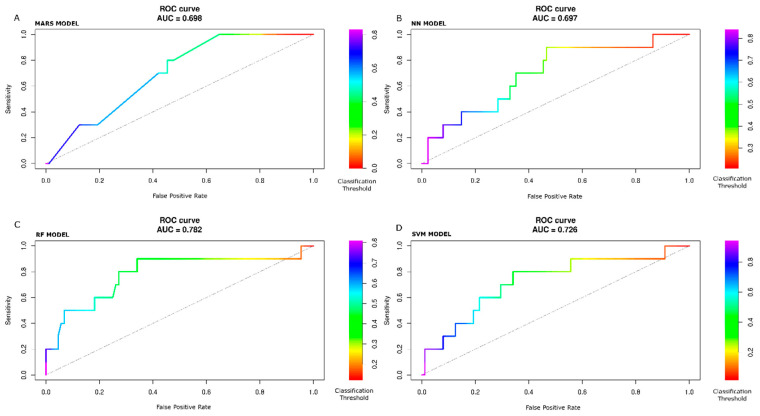
ROC curves by color scale threshold on held-out set. MARS (**A**). NN (**B**). RF (**C**). SVM (**D**) models. Right side: Color scale for different classification threshold values. The best ROC AUC value is with the RF model.

**Figure 5:**
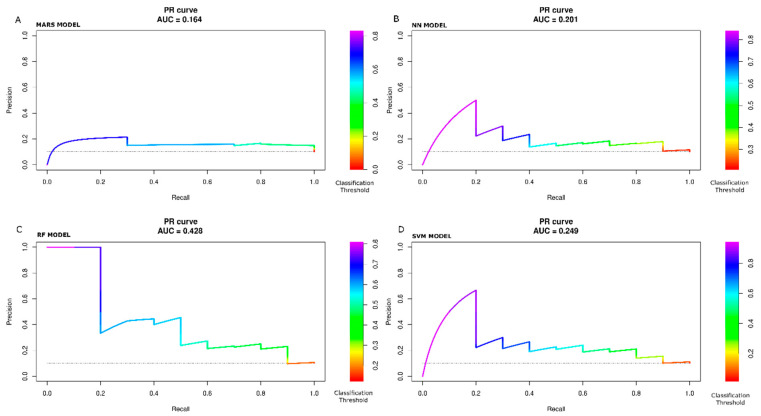
Precision-Recall (PR) prediction curve by color scale threshold on held-out set. MARS (**A**). NN (**B**). RF (**C**). SVM (**D**) models. Right side: Color scale for different classification threshold values. The best PR AUC value is with the RF model.

To consider the information of which classification threshold results in a certain point of the curves, we implemented a color scale in Figure 4A–D to show the ROC curve and in Figure 5A–D to show the PR curves with MARS, NN, RF, and SVM models, respectively. The color scale is a helpful tool to assess sensitivity variation as a function of one minus specificity (ROC curve) and precision as a function of recall (PR curve) for different classification threshold values. Typically, the metrics shown in Table 4 are calculated at the 0.50 classification threshold. The value of 0.50 represents the cut-off to decide if, during the prediction step, the patient outcome is classified to belong to one class or another (in our study, POAF or not-POAF). Of course, the value of 0.50 is not the only possible value. So the colored scale allows us to check how the metrics change as the classification cut-off changes. For example, looking at Figure 4C (RF model), with a cut-off (or threshold) of about 0.25 (yellow color), the sensitivity is about 0.9, and the false positive rate is about 0.75. With a cut-off of about 0.7 (blue color), the sensitivity is about 0.2, and the false positive rate is about 0.04. Ultimately, there is a trade-off between conflicting sensitivity and false positive rate values, and these depend on the chosen cut-off value.

With the test data set, the ROC and PR AUC values are with the MARS model, 0.70, 0.16; with the NN model, 0.70, 0.20; with the RF model, 0.78, 0.43; with the SVM model, 0.73, 0.25.

#### 3.2.4. Age-Only Models vs. All-Feature Models

In Table 1, age is significantly higher in the POAF group compared to the non-POAF group. Moreover, Figure 3A–D show that age is the most important feature (100%) selected by all four models. To prove the features were significantly predictive of POAF, we trained the models only on the age feature despite POAF’s strong age dependence. Prediction ROC AUC comparison with the models trained on other features yielded the following results (age only vs. all-feature models): MARS model, 0.51 vs. 0.70, *p*-value: 0.045; NN model, 0.50 vs. 0.70, *p*-value: 0.025; RF model, 0.52 vs. 0.78, *p*-value: 0.031; SVM model, 0.50 vs. 0.76, *p*-value: 0.045.

### 3.3. Logistic Regression

The accuracy of the logistic regression model was 64% (95% CI (54%, 74%)), and the ROC AUC value was 0.64 on the test (held-out) data set.

## 4. Discussion

This paper aimed to provide an effective prediction model of POAF following CABG and highlight the most relevant patient and clinical features selected through ML approaches. The application of ML for clinical predictions requires an accurate evaluation of the models and their hyperparameters before choosing the suitable model targeted for the specific purpose.

One of the more exciting features of our work was that none of the models reached the highest ROC, sensitivity, and specificity together. Indeed, the NN had the highest ROC and sensitivity, while RF obtained the highest specificity. Given the unbalanced nature of the dataset, PR curves were analyzed together with ROC curves, and the RF model demonstrated better performance. The RF model showed that age (100.0%), preoperative creatinine values (86.1%), time of aortic cross-clamping (82.2%), body surface area (80.9%), Logistic Euro-Score (80.7%), and extracorporeal circulation time (65.7%) were the predictors with a normalized contribution to the model greater than 40%.

Analyzing the confusion matrices of the test data, the RF model reaches the highest values of the parameters of measurement of the prediction performance, except for the sensitivity value (0.60), which is the same as the other models (the MARS model has a slightly higher value). Furthermore, with the RF model, the sensitivity and specificity values of the prediction are lower than the corresponding maximum values of the resampling, reflecting that the RF model has learned to generalize better than the other models, also considering the maximum value resampling of the ROC AUC.

The confusion matrices were calculated at the threshold value of 0.50, one of the possible referring values. Therefore, the comparative evaluation of the performance of the models cannot be based only on confusion matrices. Still, the evaluation of the ROC and PR curves are necessary for an overall classification goodness measure.

The ROC AUC analysis performed with the test data showed that RF had the highest values. Nevertheless, more than the ROC curve examination is required to represent the goodness of the prediction fully. When data are unbalanced, it is recommendable using the PR curve. As our data were moderately unbalanced, we used the PR curves to confirm the ROC curves’ feature further.

Observing the graph of the PR curve for the RF model, for threshold values from about 0.7 to 0.8, the precision is very high (equal to 1), but the recall is relatively low (between 0.0 and 0.2). As soon as the threshold value is reduced (<0.6, >0.4), the precision significantly lowers, remaining around 0.4. Nonetheless, in this setup, the recall value rises. Further decreases in the threshold value (<0.4) improve recall but worsen precision until the baseline value is reached for threshold values <0.2.

Finally, we noted that the performance of the traditional logistic regression was lower than that obtained with the RF model.

### Clinical Considerations

Prediction models for incident AF have been employed to contribute to AF screening by determining a risk category for each patient [42]. In particular, the CHARGE-AF appeared most suitable for primary screening purposes [43]. Atrial fibrillation [AF] occurs in 20% to 40% of patients after CABG [18,44,45,46,47,48]. Models have been developed to identify patients at high risk for the development of AF after CABG [49]. Nonetheless, classical statistical methods assume linear relationships between variables and predicted outcomes leading to biased results.

In this project, we propose an artificial intelligence (AI)-based prediction model to provide an effective prediction model of POAF following CABG and highlight the most relevant patient and clinical features selected through ML approaches. This is the first attempt to identify the best predictor model for future clinical application in larger population cohorts. In addition, this is the first step that will lead to implementing such a model for clinical inferences and designing a risk score to be used at the patient’s bedside.

Age is a well-known independent predictor of POAF [50,51]. This can be explained by age-related structural changes, such as increased fibrosis and atrial dilatation [52] and changes in atria’s electrophysiological properties, which predispose to the development of AF [22]. Furthermore, related comorbidities in older patients may be responsible for the increased incidence of POAF in the elderly [53]. The importance of Euro-Score confirms this as a predictor, reflecting the severe status of the patients with associated cardiovascular and non-cardiovascular morbidities [54].

In our model, extracorporeal circulation time (ECC) and cardiopulmonary bypass time (CPB) are significantly related to POAF. CPB has been associated with an ischemia-reperfusion injury-inducing a complex inflammatory response, which has been reported in patients with AF. These range from inflammatory infiltrates in atrial biopsies to increased concentrations of C-reactive protein, which form the substrate for the generation of ectopic activity [55,56]. Nonetheless, it is still controversial whether CABG performed on the beating heart without ECC and CC reduces the incidence of POAF [54].

The mechanism of how POAF is influenced by low renal function has yet to be fully understood. Nonetheless, the increased incidence of hypertension, fluid overload, and pathological activation of the intrarenal renin–angiotensin–aldosterone might explain this association [57]. In addition, renal dysfunction was associated with both electrical and structural remodeling of LA, which might be the mechanism underlying the pathophysiology of new-onset POAF [58].

Finally, body surface area was an independent risk factor for new-onset AF, confirming previous reports [59,60]. Other studies have shown that BSA is only a risk factor for POAF in older patients [61]. Increased left atrium stretch, diastolic dysfunction [62], and high plasma volume secondary to obesity [63] have been proposed as mechanisms for the vulnerability of the left atrium to the development of POAF.

## 5. Limitations

The study presents some inherent limitations that need to be highlighted.

First, the predictive models were not tested and validated on cohorts from other centers. Second, the hyperparameters search was limited by available hardware. Third, the small number of patients likely reduces the prediction capacity of the trained models. However, we preferred testing our models on actual clinical data accepting a limited cohort. Fourth, the initial data set might include only some AF predictive variables; more risk factors can lead to more precise models. Nonetheless, this was the first attempt toward an upcoming accurate score model based on ML. Finally, the relatively low level of ROC AUC obtained could be due to the low number of cases or the available variables’ low prediction capacity. Alternatively, it could be due to the limited refinement of the hyperparameters. However, this limitation is shared with many previously published papers employing actual data.

In addition, the lack of ECG data represents a further limitation. We are working on ECG-ML-reading procedures that will be the objective of a forthcoming paper. Finally, we should have carried on external validation datasets to prove the generalizability of the models.

Finally, we did not explore whether POAF persisted beyond the discharge from the hospital. A machine learning analysis of who persists in POAF after CABG, despite rhythm control, would be very interesting, and it is a call for further research.

## 6. Conclusions

Random Forest is best performed in the clinical prediction of postoperative atrial fibrillation following coronary artery bypass grafting. The ML technique is promising for more sophisticated and accurate AI-based risk score models in this setting.

Further research employing other ML methods and more observations is warranted to yield more accurate ML predictive performance.

## Figures and Tables

**Table 1:** Descriptive characteristics.

	Overall	No POAF	POAF	*p*
	(*n* = 394)	(*n* = 352)	(*n* = 42)	
Female/Male	60/334 (15.2/84.8)	53/299 (15.1/84.9)	7/35 (16.7/83.3)	0.962
Age	60.00 [54.00, 67.00]	59.00 [53.00, 66.00]	66.00 [59.25, 69.00]	<0.001
BSA (m^2^)	2.00 [1.88, 2.12]	2.00 [1.88, 2.12]	2.03 [1.90, 2.13]	0.489
Hypertension	265 (67.3)	237 (67.3)	28 (66.7)	1
Type II DM	166 (42.1)	148 (42.0)	18 (42.9)	1
Hyperlipidemia	196 (49.7)	177 (50.3)	19 (45.2)	0.625
COPD	38 (9.6)	34 (9.7)	4 (9.5)	1
CKD	88 (22.3)	79 (22.4)	9 (21.04)	1
TIA/Stroke	22 (5.6)	19 (5.4)	3 (7.1)	0.718
PVD	54 (13.7)	49 (13.9)	5 (11.9)	1
Year				0.122
2010	10 (2.5)	9 (2.6)	1 (2.4)	
2011	8 (2.0)	8 (2.3)	0 (0.0)	
2012	22 (5.6)	20 (5.7)	2 (4.8)	
2013	67 (17.0)	66 (18.8)	1 (2.4)	
2014	99 (25.1)	87 (24.7)	12 (28.6)	
2015	72 (18.3)	59 (16.8)	13 (31.0)	
2016	76 (19.3)	67 (19.0)	9 (21.4)	
2017	40 (10.2)	36 (10.2)	4 (9.5)	
Preoperative Creatinine *	85.00 [76.00, 96.00]	84.00 [75.00, 96.00]	87.00 [78.00, 97.00]	0.387
Euro-Score (Log)	1.55 [1.11, 2.66]	1.52 [1.07, 2.64]	1.66 [1.34, 3.39]	0.081
Status				0.363
Elective	151 (38.3)	134 (38.1)	17 (40.5)	
Emergency	14 (3.6)	11 (3.1)	3 (7.1)	
Urgency	229 (58.1)	207 (58.8)	22 952.4)	
OR Risk				0.013
high	36 (9.1)	27 (7.7)	9 (21.4)	
low	308 (78.2)	279 (79.3)	29 (69.0)	
medium	50 (12.7)	46 (13.1)	4 (9.5)	
CABG/OPCAB	372/22 (94.4/5.6)	333/19 (94.6/5.4)	39/3 (92.9/7.1)	0.912
Proximal Anastomoses				0.884
1	81 (20.6)	73 (20.7)	8 (19.0)	
2	14 (3.6)	12 (3.4)	2 (4.8)	
Distal Anastomoses				0.281
2	90 (22.8)	77 (21.9)	13 (31.0)	
3	178 (45.2)	158 (44.9)	20 (47.6)	
4	101 (25.6)	95 (27.0)	6 (14.3)	
5	25 (6.3)	22 (6.2)	3 (7.1)	
ECC Time (min)	81 [65, 100]	82 [64.25, 100]	75 [67.5, 87.5]	0.280
Clamping Time	59 [45, 74.25]	60 [44, 75]	57 [49.5, 69]	0.664
Complications				0.534
1	39 (9.9)	37 (10.5)	2 (4.8)	
2	5 (1.3)	4 (1.1)	1 (2.4)	
3	3 (0.8)	3 (0.9)	0 (0.0)	
EC	-	-	2 (4.8)	
FC	-	-	12 (28.5)	
EC + FC	-	-	27 (64.3)	

Values are expressed as *n* (%) with categorical variables or median (Q1, Q3) with continuous variables. Abbreviations: POAF: Postoperative Atrial Fibrillation; BSA: Body Surface Area; DM: Diabetes Mellitus; COPD: Chronic Obstructive Pulmonary Disease; CKD: Chronic Kidney Disease; TIA: Transient Ischemic Attack; PVD: Peripheral Vascular Disease; CABG: Coronary Artery Bypass Graft; OPCAB: Off-Pump Coronary Artery Bypass; BIMA: Bilateral Internal Mammary Artery; OR: Operative Risk Score; ECC: Extra Corporeal Circulation; EC: Electric Cardioversion; FC: Pharmacological Cardioversion. * Micromoles/L.

**Table 2:** Maximum ROC AUC, Sensitivity, and Specificity Values with each Model.

	Max.
ROC AUC	
MARS	0.87
NN	0.94
RF	0.78
SVM	0.95
Sensitivity	
MARS	1
NN	1
RF	0.67
SVM	1
Specificity	
MARS	0.74
NN	1
RF	0.89
SVM	0.74

Abbreviations: ROC: Receiver Operating Characteristic; AUC: Area Under Curve; MARS: Multivariate Adaptive Regression Spline; NN: Neural Network; RF: Random Forest; SVM: Support Vector Machine.

**Table 3:** Estimated Differences Between Models.

	ROC AUC	*p*-Value	Sensitivity	*p*-Value	Specificity	*p*-Value
RF—SVM	−0.06	0.42	−0.19	0.16	0.12	0.05
NN—SVM	0.10	1.00	0.31	0.35	−0.28	0.26
NN—RF	0.16	0.18	0.50	0.03	−0.40	0.06
MARS—SVM	−0.02	1.00	0.07	1.00	−0.06	1.00
MARS—RF	0.05	1.00	0.26	0.26	−0.19	0.05
MARS—NN	−0.11	0.52	−0.24	0.85	0.22	0.61

Abbreviations: ROC: Receiver Operating Characteristic; AUC: Area Under Curve; MARS: Multivariate Adaptive Regression Spline; NN: Neural Network; RF: Random Forest; SVM: Support Vector Machine.

**Table 4:** Predicted vs. True Outcome Values Statistics (at 0.50 classification threshold).

Metrics	MARS	NN	RF	SVM
Accuracy [95% CI]	0.58 [0.48–0.68]	0.66 [0.56–0.76]	0.79 [0.69–0.86]	0.69 [0.59–0.78]
Sensitivity or Recall	0.70	0.60	0.60	0.60
Specificity	0.57	0.67	0.81	0.70
Precision or Positive Predicted Value	0.16	0.17	0.26	0.19
Negative Predicted Value	0.94	0.94	0.95	0.94
F1	0.25	0.27	0.36	0.29

Abbreviations: MARS: Multivariate Adaptive Regression Spline; NN: Neural Network; RF: Random Forest; SVM: Support Vector Machine; CI: Confidence Interval; F1: Harmonic mean between precision and recall.

## Data Availability

The data presented in this study are available on request from the corresponding author. The data are not publicly available due to GDPR privacy restrictions.

## Supplementary Materials

The following are available online at https://www.mdpi.com/article/10.3390/jcdd10020082/s1; Table S1. Training dataset descriptive characteristics; Table S2. Test dataset descriptive characteristics.

## References

[1] Chugh S.S., Havmoeller R., Narayanan K., Singh D., Rienstra M., Benjamin E.J., Gillum R.F., Kim Y.H., McAnulty J.H., Zheng Z.J. (2014). Worldwide Epidemiology of Atrial Fibrillation: A Global Burden of Disease 2010 Study. Circulation.

[2] Go A.S., Hylek E.M., Phillips K.A., Chang Y., Henault L.E., Selby J.V., Singer D.E. (2001). Prevalence of Diagnosed Atrial Fibrillation in Adults: National Implications for Rhythm Management and Stroke Prevention: The Anticoagulation and Risk Factors in Atrial Fibrillation [ATRIA) Study. JAMA.

[3] Zoni-Berisso M., Lercari F., Carazza T., Domenicucci S. (2014). Epidemiology of atrial fibrillation: European perspective. Clin. Epidemiol..

[4] Schnabel R.B., Yin X., Gona P., Larson M.G., Beiser A.S., McManus D.D., Newton-Cheh C., A Lubitz S., Magnani J.W., Ellinor P.T. (2015). 50 year trends in atrial fibrillation prevalence, incidence, risk factors, and mortality in the Framingham Heart Study: A cohort study. Lancet.

[5] Lancaster T.S., Melby S.J., Damiano R.J. (2016). Minimally invasive surgery for atrial fibrillation. Trends Cardiovasc. Med..

[6] Roten L., Derval N., Pascale P., Scherr D., Komatsu Y., Shah A., Ramoul K., Denis A., Sacher F., Hocini M. (2012). Current Hot Potatoes in Atrial Fibrillation Ablation. Curr. Cardiol. Rev..

[7] Schuessler R.B., Lee A.M., Melby S.J., Voeller R.K., Gaynor S.L., Sakamoto S.-I., Damiano R.J. (2009). Animal studies of epicardial atrial ablation. Heart Rhythm..

[8] Oral H., Knight B.P., Tada H., Özaydın M., Chugh A., Hassan S., Scharf C., Lai S.W., Greenstein R., Pelosi F. (2002). Pulmonary vein isolation for paroxysmal and persistent atrial fibrillation. Circulation.

[9] Opolski G., Torbicki A., Kosior D.A., Szulc M., Wozakowska-Kapłon B., Kołodziej P., Achremczyk P. (2004). Rate control vs rhythm control in patients with nonvalvular persistent atrial fibrillation: The results of the Polish How to Treat Chronic Atrial Fibrillation [HOT I) Study. Chest.

[10] La Meir M., Gelsomino S., Lucà F., Pison L., Colella A., Lorusso R., Crudeli E., Gensini G.F., Crijns H.G., Maessen J. (2013). Minimal invasive surgery for atrial fibrillation: An updated review. Europace.

[11] Ringborg A., Nieuwlaat R., Lindgren P., Jönsson B., Fidan D., Maggioni A.P., Lopez-Sendon J., Stepinska J., Cokkinos D.V., Crijns H.J. (2008). Costs of atrial fibrillation in five European countries: Results from the Euro Heart Survey on atrial fibrillation. Europace.

[12] Di Carlo A., Bellino L., Consoli D., Mori F., Zaninelli A., Baldereschi M., Cattarinussi A., D’Alfonso M.G., Gradia C., Sgherzi B. (2019). Prevalence of atrial fibrillation in the Italian elderly population and projections from 2020 to 2060 for Italy and the European Union: The FAI Project. Europace.

[13] Akoum N., Daccarett M., McGann C., Segerson N., Vergara G., Kuppahally S., Badger T., Burgon N., Haslam T., Kholmovski E. (2010). Atrial Fibrosis Helps Select the Appropriate Patient and Strategy in Catheter Ablation of Atrial Fibrillation: A DE-MRI Guided Approach. J. Cardiovasc. Electrophysiol..

[14] Sacher F., Wright M., Tedrow U.B., O’Neill M., Jais P., Hocini M., Macdonald R., Davies D.W., Kanagaratnam P., Derval N. (2010). Wolff-Parkinson-White ablation after a prior failure: A 7-year multicentre experience. Europace.

[15] Benjamin E.J., Muntner P., Alonso A., Bittencourt M.S., Callaway C.W., Carson A.P., Chamberlain A.M., Chang A.R., Cheng S., Das S.R. (2019). Heart Disease and Stroke Statistics-2019 Update: A Report from the American Heart Association. Circulation.

[16] Brüggenjürgen B., Rossnagel K., Roll S., Andersson F.L., Selim D., Müller-Nordhorn J., Nolte C.H., Jungehülsing G.J., Villringer A., Willich S.N. (2007). The Impact of Atrial Fibrillation on the Cost of Stroke: The Berlin Acute Stroke Study. Value Health.

[17] Winter Y., Wolfram C., Schaeg M., Reese J.-P., Oertel W.H., Dodel R., Back T. (2009). Evaluation of costs and outcome in cardioembolic stroke or TIA. J. Neurol..

[18] Hindricks G., Potpara T., Dagres N., Arbelo E., Bax J.J., Blomström-Lundqvist C., Boriani G., Castella M., Dan G.A., Dilaveris P.E. (2021). Corrigendum to 2020 ESC Guidelines for the diagnosis and management of atrial fibrillation developed in collaboration with the European Association for Cardio-Thoracic Surgery [EACTS): The Task Force for the diagnosis and management of atrial fibrillation of the European Society of Cardiology [ESC) Developed with the special contribution of the European Heart Rhythm Association [EHRA) of the ESC. Eur. Heart J..

[19] Echahidi N., Pibarot P., O’Hara G., Mathieu P. (2008). Mechanisms, Prevention, and Treatment of Atrial Fibrillation After Cardiac Surgery. J. Am. Coll. Cardiol..

[20] Filardo G., Damiano R.J., Ailawadi G., Thourani V.H., Pollock B.D., Sass D.M., Phan T.K., Nguyen H., Da Graca B. (2018). Epidemiology of new-onset atrial fibrillation following coronary artery bypass graft surgery. Heart.

[21] Almassi G.H., Schowalter T., Nicolosi A.C., Aggarwal A., Moritz T.E., Henderson W.G., Tarazi R., Shroyer A.L., Sethi G.K., Grover F.L. (1997). Atrial Fibrillation after Cardiac Surgery: A Major Morbid Event?. Ann. Surg..

[22] Mathew J.P., Fontes M.L., Tudor I.C., Ramsay J., Duke P., Mazer C.D., Barash P.G., Hsu P.H., Mangano D.T. (2004). A Multicenter Risk Index for Atrial Fibrillation after Cardiac Surgery. JAMA.

[23] Kalavrouziotis D., Buth K.J., Ali I.S. (2007). The Impact of New-Onset Atrial Fibrillation on In-hospital Mortality Following Cardiac Surgery. Chest.

[24] LaPar D.J., Speir A.M., Crosby I.K., Fonner E., Brown M., Rich J.B., Quader M., Kern J.A., Kron I.L., Ailawadi G. (2014). Postoperative Atrial Fibrillation Significantly Increases Mortality, Hospital Readmission, and Hospital Costs. Ann. Thorac. Surg..

[25] Aranki S.F., Shaw D.P., Adams D.H., Rizzo R.J., Couper G.S., VanderVliet M., Collins J.J., Cohn L.H., Burstin H.R. (1996). Predictors of Atrial Fibrillation after Coronary Artery Surgery. Circulation.

[26] Hill N.R., Ayoubkhani D., McEwan P., Sugrue D.M., Farooqui U., Lister S., Lumley M., Bakhai A., Cohen A.T., O’Neill M. (2019). Predicting atrial fibrillation in primary care using machine learning. PLoS ONE.

[27] Deo R.C. (2015). Machine Learning in Medicine. Circulation.

[28] Ren X., Mi Z., Georgopoulos P.G. (2020). Comparison of Machine Learning and Land Use Regression for fine scale spatiotemporal estimation of ambient air pollution: Modeling ozone concentrations across the contiguous United States. Environ. Int..

[29] Tseng A.S., Noseworthy P.A. (2021). Prediction of Atrial Fibrillation Using Machine Learning: A Review. Front. Physiol..

[30] Bidar E., Bramer S., Maesen B., Maessen J.G., Schotten U. (2013). Post-operative Atrial Fibrillation-Pathophysiology, Treatment and Prevention. J. Atr. Fibrillation.

[31] Camm A.J., Kirchhof P., Lip G.Y., Schotten U., Savelieva I., Ernst S., Van Gelder I.C., Al-Attar N., Hindricks G., Developed with the Special Contribution of the European Heart Rhythm Association (EHRA) (2010). Guidelines for the management of atrial fibrillation: The Task Force for the Management of Atrial Fibrillation of the European Society of Cardiology (ESC). Eur. Heart J..

[32] Konings K.T., Kirchhof C.J., Smeets J.R., Wellens H.J., Penn O.C., Allessie M.A. (1994). High-density mapping of electrically induced atrial fibrillation in humans. Circulation.

[33] Kuhn M. (2008). Building Predictive Models in *R* Using the caret Package. J. Stat. Soft.

[34] Tetta C., Moula A.I., Matteucci F., Parise O., Maesen B., Johnson D., La Meir M., Gelsomino S. (2019). Association between atrial fibrillation and Helicobacter pylori. Clin. Res. Cardiol..

[35] Matteucci F., Maesen B., De Asmundis C., Parise G., Micali L.R., Tuijthof G., Gerits P., Vernooy K., Maessen J.G., La Meir M. (2021). New Biparietal Bipolar Catheter Prototype for Hybrid Atrial Fibrillation Ablation. Innovations.

[36] Gharaviri A., Bidar E., Potse M., Zeemering S., Verheule S., Pezzuto S., Krause R., Maessen J.G., Auricchio A., Schotten U. (2020). Epicardial Fibrosis Explains Increased Endo–Epicardial Dissociation and Epicardial Breakthroughs in Human Atrial Fibrillation. Front. Physiol..

[37] Gharaviri A., Verheule S., Eckstein J., Potse M., Kuijpers N.H., Schotten U. (2012). A computer model of endo-epicardial electrical dissociation and transmural conduction during atrial fibrillation. Europace.

[38] Gharaviri A., Pezzuto S., Potse M., Conte G., Zeemering S., Sobota V., Verheule S., Krause R., Auricchio A., Schotten U. (2021). Synergistic antiarrhythmic effect of inward rectifier current inhibition and pulmonary vein isolation in a 3D computer model for atrial fibrillation. Europace.

[39] Frix A.-N., Cousin F., Refaee T., Bottari F., Vaidyanathan A., Desir C., Vos W., Walsh S., Occhipinti M., Lovinfosse P. (2021). Radiomics in Lung Diseases Imaging: State-of-the-Art for Clinicians. J. Pers. Med..

[40] Zerka F., Urovi V., Bottari F., Leijenaar R.T., Walsh S., Gabrani-Juma H., Gueuning M., Vaidyanathan A., Vos W., Occhipinti M. (2021). Privacy preserving distributed learning classifiers—Sequential learning with small sets of data. Comput. Biol. Med..

[41] Wu G., Yang P., Xie Y., Woodruff H.C., Rao X., Guiot J., Frix A.N., Louis R., Moutschen M., Li J. (2020). Development of a clinical decision support system for severity risk prediction and triage of COVID-19 patients at hospital admission: An international multicentre study. Eur. Respir. J..

[42] Moons K.G., Kengne A.P., Woodward M., Royston P., Vergouwe Y., Altman D.G., Grobbee D.E. (2012). Risk prediction models: I. Development, internal validation, and assessing the incremental value of a new (bio)marker. Heart.

[43] Himmelreich J.C.L., Veelers L., Lucassen W.A.M., Schnabel R.B., Rienstra M., van Weert H.C.P.M., E Harskamp R. (2020). Prediction models for atrial fibrillation applicable in the community: A systematic review and meta-analysis. Europace.

[44] D’Agostino R.S., Jacobs J.P., Badhwar V., Fernandez F.G., Paone G., Wormuth D.W., Shahian D.M. (2018). The Society of Thoracic Surgeons Adult Cardiac Surgery Database: 2018 Update on Outcomes and Quality. Ann. Thorac. Surg..

[45] Shen J., Lall S., Zheng V., Buckley P., Damiano R.J., Schuessler R.B. (2011). The persistent problem of new-onset postoperative atrial fibrillation: A single-institution experience over two decades. J. Thorac. Cardiovasc. Surg..

[46] D’Agostino R.S., Jacobs J.P., Badhwar V., Paone G., Rankin J.S., Han J.M., McDonald D., Shahian D.M. (2016). The Society of Thoracic Surgeons Adult Cardiac Surgery Database: 2016 Update on Outcomes and Quality. Ann. Thorac. Surg..

[47] Philip I., Berroëta C., Leblanc I. (2014). Perioperative challenges of atrial fibrillation. Curr. Opin. Anaesthesiol..

[48] Frendl G., Sodickson A.C., Chung M.K., Waldo A.L., Gersh B.J., Tisdale J.E., Calkins H., Aranki S., Kaneko T., Cassivi S. (2014). 2014 AATS guidelines for the prevention and management of perioperative atrial fibrillation and flutter for thoracic surgical procedures. J. Thorac. Cardiovasc. Surg..

[49] Fan K., Chen L., Liu F., Ding X., Yan P., Gao M., Yu W., Liu H., Yu Y. (2022). Predicting New-Onset Postoperative Atrial Fibrillation Following Isolated Coronary Artery Bypass Grafting: Development and Validation of a Novel Nomogram. Int. J. Gen. Med..

[50] Haghjoo M., Basiri H., Salek M., Sadr-Ameli M.A., Kargar F., Raissi K., Omrani G., Tabatabaie M.B., Sadeghi H.M., Tabaie A.S. (2008). Predictors of Postoperative Atrial Fibrillation after Coronary Artery Bypass Graft Surgery. Indian Pacing Electrophysiol. J..

[51] Borde D., Gandhe U., Hargave N., Pandey K., Mathew M., Joshi S. (2014). Prediction of postoperative atrial fibrillation after coronary artery bypass grafting surgery: Is CHA 2 DS 2 -VASc score useful?. Ann. Card. Anaesth..

[52] Davies M.J., Pomerance A. (1972). Pathology of atrial fibrillation in man. Br. Heart J..

[53] Cox J.L., Boineau J.P., Schuessler R.B., Kater K.M., Lappas D.G. (1993). Five-year experience with the maze procedure for atrial fibrillation. Ann. Thorac. Surg..

[54] Ghurram A., Krishna N., Bhaskaran R., Kumaraswamy N., Jayant A., Varma P.K. (2020). Patients who develop post-operative atrial fibrillation have reduced survival after off-pump coronary artery bypass grafting. Indian J. Thorac. Cardiovasc. Surg..

[55] Archbold R., Schilling R. (2004). Atrial pacing for the prevention of atrial fibrillation after coronary artery bypass graft surgery: A review of the literature. Heart.

[56] Hogue C.W., Domitrovich P.P., Stein P.K., Despotis G.D., Re L., Schuessler R.B., Kleiger R.E., Rottman J.N. (1998). RR interval dynamics before atrial fibrillation in patients after coronary artery bypass graft surgery. Circulation.

[57] Soliman E.Z., Prineas R.J., Go A.S., Xie D., Lash J.P., Rahman M., Ojo A., Teal V.L., Jensvold N.G., Robinson N.L. (2010). Chronic kidney disease and prevalent atrial fibrillation: The Chronic Renal Insufficiency Cohort (CRIC). Am. Heart J..

[58] Chang S.-L., Chen Y.-C., Chen Y.-J., Wangcharoen W., Lee S.-H., Lin C.-I., Chen S.-A. (2007). Mechanoelectrical feedback regulates the arrhythmogenic activity of pulmonary veins. Heart.

[59] Wang T.J., Parise H., Levy D., D’Agostino R.B., Wolf P.A., Vasan R.S., Benjamin E.J. (2004). Obesity and the risk of new-onset atrial fibrillation. JAMA.

[60] Zacharias A., Schwann T.A., Riordan C.J., Durham S.J., Shah A.S., Habib R.H. (2005). Obesity and Risk of New-Onset Atrial Fibrillation After Cardiac Surgery. Circulation.

[61] Echahidi N., Mohty D., Pibarot P., Després J.-P., O’Hara G., Champagne J., Philippon F., Daleau P., Voisine P., Mathieu P. (2007). Obesity and Metabolic Syndrome Are Independent Risk Factors for Atrial Fibrillation After Coronary Artery Bypass Graft Surgery. Circulation.

[62] Tsang T.S.M., Barnes M.E., Gersh B.J., Bailey K.R., Seward J.B. (2002). Left atrial volume as a morphophysiologic expression of left ventricular diastolic dysfunction and relation to cardiovascular risk burden. Am. J. Cardiol..

[63] Iacobellis G., Ribaudo M.C., Leto G., Zappaterreno A., Vecci E., Di Mario U., Leonetti F. (2002). Influence of Excess Fat on Cardiac Morphology and Function: Study in Uncomplicated Obesity. Obes. Res..

