# Supplementary material for: Machine Learning to Identify Patients at Risk of Developing New-Onset Atrial Fibrillation after Coronary Artery Bypass
Source: J Cardiovasc Dev Dis. 2023 Feb 15;10(2):82. doi: 10.3390/jcdd10020082 (PMC9962068; doi:10.3390/jcdd10020082)
Supplement: Supplementary file 1 [file jcdd-10-00082-s001.zip › jcdd-2109800-supplementary.pdf]

Table S1. Training dataset descriptive characteristics.				
	**Overall**	**No POAF**	**POAF**	**p**
	**(n=296)**	**(n=264)**	**(n=32)**	
**Female/Male**	48/248 (16.2/83.8)	41/223 (15.5/84.5)	7/25 (21.9/78.1)	0.506
**Age**	0.50 [0.37, 0.61]	0.48 [0.36, 0.61]	0.54 [0.46, 0.66]	0.006
**BSA (m^2)**	0.53 [0.40, 0.64]	0.52 [0.40, 0.64]	0.55 [0.42, 0.65]	0.553
**Hypertension**	198 (67)	177 (67)	21 (66)	0.845
**Type II DM**	125 (42)	111 (42)	14 (44)	0.852
**Hyperlipidemia**	146 (49)	132 (50)	14 (44)	0.576
**COPD**	29 (10)	26 (10)	3 (9)	1
**CKD**	68 (23)	61 (23)	7 (22)	1
**TIA/Stroke**	12 (4)	10 (4)	2 (6)	0.626
**PVD**	42 (14)	38 (14)	4 (12)	1
**Year**				
**2010**	9 (3.0)	8 (3.0)	1 (3.1)	1
**2011**	6 (2.0)	6 (2.3)	0 (0.0)	0.843
**2012**	13 (4.4)	11 (4.2)	2 (6.2)	0.931
**2013**	49 (16.6)	48 (18.2)	1 (3.1)	0.056
**2014**	78 (26.4)	69 (26.1)	9 (28.1)	0.977
**2015**	52 (17.6)	43 (16.3)	9 (28.1)	0.157
**2016**	57 (19.3)	51 (19.3)	6 (18.8)	1
**2017**	32 (10.8)	28 (10.6)	4 (12.5)	0.981
**Pre-operative Creatinine***	0.19 [0.15, 0.23]	0.19 [0.15, 0.23]	0.21 [0.16, 0.24]	0.361
**EuroScore (Log)**	0.02 [0.01, 0.05]	0.02 [0.00, 0.05]	0.02 [0.01, 0.07]	0.17
**Status**				
**Elective**	116 (39.2)	102 (38.6)	14 (43.8)	0.713
**Emergency**	10 (3.4)	8 (3.0)	2 (6.2)	0.664
**Urgency**	170 (57.4)	154 (58.3)	16 (50.0)	0.477
**OR Risk**				
**high**	28 (9.5)	21 (8.0)	7 (21.9)	0.026
**low**	230 (77.7)	209 (79.2)	21 (65.6)	0.13
**medium**	38 (12.8)	34 (12.9)	4 (12.5)	1
**CABG**	281 (94.9)	251 (95.1)	30 (93.8)	1
**OPCAB**	15 (5.1)	13 (4.9)	2 (6.2)	1
**Proximal Anastomoses**				0.777
**1**	57 (19.3)	50 (18.9)	7 (21.9)	
**2**	13 (4.4)	11 (4.2)	2 (6.2)	
**Distal Anastomoses**				0.29
**2**	65 (22.0)	55 (20.8)	10 (31.2)	
**3**	134 (45.3)	118 (44.7)	16 (50.0)	
**4**	79 (26.7)	74 (28.0)	5 (15.6)	
**5**	18 (6.1)	17 (6.4)	1 (3.1)	
**ECC Time (min)**	0.37 [0.29, 0.45]	0.37 [0.29, 0.45]	0.34 [0.28, 0.40]	0.4
**Clamping Time**	0.44 [0.31, 0.55]	0.44 [0.31, 0.56]	0.41 [0.36, 0.48]	0.498
**Complications**				0.685
**1**	28 (9.5)	26 (9.8)	2 (6.2)	
**2**	4 (1.4)	3 (1.1)	1 (3.1)	
**3**	2 (0.7)	2 (0.8)	0 (0.0)	
**EC**	-	-	1 (3.1)	

**FC**	-	-	9 (28.1)
**EC + FC**	-	-	21 (65.6)

Values are expressed as n (%) with categorical variables or median [Q1, Q3] with continuous variables. Abbreviations: POAF: Postoperative Atrial Fibrillation; BSA: Body Surface Area; DM: Diabetes Mellitus; COPD: Chronic Obstructive Pulmonary Disease; CKD: Chronic Kidney Disease; TIA: Transient Ischemic Attack; PVD: Peripheral Vascular Disease; CABG: Coronary Artery Bypass Graft; OPCAB: Off-Pump Coronary Artery Bypass; BIMA: Bilateral Internal Mammary Artery; OR: Operative Risk Score; ECC: Extra Corporeal Circulation; EC: Electric Cardioversion; FC: Pharmacological Cardioversion. *Micromoles/L.

Table S2. Test dataset descriptive characteristics.				
	Overall	No POAF	POAF	p
	(n=98)	(n=88)	(n=10)	
**Female/Male**	12/86 (12.2/87.8)	12/76 (13.6/86.4)	0/10 (0.0/100.0)	0.461
**Age**	0.46 [0.38, 0.57]	0.45 [0.36, 0.57]	0.61 [0.52, 0.62]	0.008
**BSA (m^2)**	0.52 [0.42, 0.64]	0.51 [0.41, 0.64]	0.54 [0.51, 0.64]	0.694
**Hypertension**	67 (68)	60 (68)	7 (70)	1
**Type II DM**	41 (42)	37 (42)	4 (40)	1
**Hyperlipidemia**	50 (51)	45 (51)	5 (50)	1
**COPD**	9 (9)	8 (9)	1 (10)	1
**CKD**	20 (20)	18 (20)	2 (20)	1
**TIA/Stroke**	10 (10)	9 (10)	1 (10)	1
**PVD**	12 (12)	11 (12)	1 (10)	1
**Year**				
**2010**	1 (1.0)	1 (1.1)	0 (0.0)	1
**2011**	2 (2.0)	2 (2.3)	0 (0.0)	1
**2012**	9 (9.2)	9 (10.2)	0 (0.0)	0.629
**2013**	18 (18.4)	18 (20.5)	0 (0.0)	0.249
**2014**	21 (21.4)	18 (20.5)	3 (30.0)	0.771
**2015**	20 (20.4)	16 (18.2)	4 (40.0)	0.227
**2016**	19 (19.4)	16 (18.2)	3 (30.0)	0.636
**2017**	8 (8.2)	8 (9.1)	0 (0.0)	0.7
**Pre-operative Creatinine***	0.20 [0.16, 0.24]	0.20 [0.16, 0.24]	0.21 [0.18, 0.25]	0.391
**EuroScore (Log)**	0.02 [0.01, 0.05]	0.02 [0.01, 0.05]	0.02 [0.02, 0.04]	0.337
**Status**				
**Elective**	35 (35.7)	32 (36.4)	3 (30.0)	0.96
**Emergency**	4 (4.1)	3 (3.4)	1 (10.0)	0.877
**Urgency**	59 (60.2)	53 (60.2)	6 (60.0)	1
**OR Risk**				
**high**	8 (8.2)	6 (6.8)	2 (20.0)	0.405
**low**	78 (79.6)	70 (79.5)	8 (80.0)	1
**medium**	12 (12.2)	12 (13.6)	0 (0.0)	0.461
**CABG**	91 (92.9)	82 (93.2)	9 (90.0)	1
**OPCAB**	7 (7.1)	6 (6.8)	1 (10.0)	1
**Proximal Anastomoses**				0.49
**1**	24 (24.5)	23 (26.1)	1 (10.0)	
**2**	1 (1.0)	1 (1.1)	0 (0.0)	
**Distal Anastomoses**				0.322
**2**	25 (25.5)	22 (25.0)	3 (30.0)	
**3**	44 (44.9)	40 (45.5)	4 (40.0)	
**4**	22 (22.4)	21 (23.9)	1 (10.0)	
**5**	7 (7.1)	5 (5.7)	2 (20.0)	
**ECC Time (min)**	0.34 [0.27, 0.45]	0.34 [0.28, 0.46]	0.34 [0.26, 0.36]	0.522
**Clamping Time**	0.40 [0.30, 0.56]	0.39 [0.30, 0.56]	0.44 [0.33, 0.50]	0.869
**Complications**				0.636
**1**	11 (11.2)	11 (12.5)	0 (0.0)	
**2**	1 (1.0)	1 (1.1)	0 (0.0)	
**3**	1 (1.0)	1 (1.1)	0 (0.0)	
**EC**	-	-	1 (10.0)	

**FC**	-	-	3 (30.0)
**EC + FC**	-	-	6 (60.0)

Values are expressed as n (%) with categorical variables or median [Q1, Q3] with continuous variables. Abbreviations: POAF: Postoperative Atrial Fibrillation; BSA: Body Surface Area; DM: Diabetes Mellitus; COPD: Chronic Obstructive Pulmonary Disease; CKD: Chronic Kidney Disease; TIA: Transient Ischemic Attack; PVD: Peripheral Vascular Disease; CABG: Coronary Artery Bypass Graft; OPCAB: Off-Pump Coronary Artery Bypass; BIMA: Bilateral Internal Mammary Artery; OR: Operative Risk Score; ECC: Extra Corporeal Circulation; EC: Electric Cardioversion; FC: Pharmacological Cardioversion. *Micromoles/L.
